# High prevalence of gestational night blindness and maternal anemia in a population-based survey of Brazilian Amazonian postpartum women

**DOI:** 10.1371/journal.pone.0219203

**Published:** 2019-07-03

**Authors:** Paulo A. R. Neves, Bárbara H. Lourenço, Anaclara Pincelli, Maíra B. Malta, Rodrigo M. Souza, Marcelo U. Ferreira, Marcia C. Castro, Marly A. Cardoso, for the MINA-Brazil Study Group

**Affiliations:** 1 Department of Nutrition, School of Public Health, University of São Paulo, São Paulo, SP, Brazil; 2 Department of Parasitology, Institute of Biomedical Sciences, University of São Paulo, São Paulo, SP, Brazil; 3 Multidisciplinary Center, Federal University of Acre, Cruzeiro do Sul, Brazil; 4 Department of Global Health and Population, Harvard T.H. Chan School of Public Health, Boston, MA, United States of America; University of Ghana, GHANA

## Abstract

Nutrition during pregnancy is one of the key elements to good maternal and child health, as well as to lifetime landmarks. However, many pregnant women go undernourished in less developed settings. The purpose of this study was to estimate the prevalence and factors associated with gestational night blindness (GXN) and maternal anemia in a cross-sectional population-based study in Cruzeiro do Sul, Acre State, Western Brazilian Amazon. All women living in the municipality admitted at the only maternity-hospital in the city to delivery of a singleton infant were eligible to this study (n = 1,525). Recruitment of participants took place between July 2015 to June 2016. GXN was assessed in the postpartum period by WHO standardized interview. Maternal anemia was defined as hemoglobin at delivery < 110.0 g/L. We estimated prevalence rates and adjusted prevalence ratios (aPR), alongside 95% confidence intervals (95% CI), of the factors associated with the outcomes through multiple Poisson regression models with robust variance. Alarming prevalence of GXN (11.5%; 95% CI, 9.97–13.25) and maternal anemia (39.4%; 95% CI, 36.84–41.95) were found. Factors associated with GXN were (aPR; 95% CI): ≥ 5 residents in the household (2.06; 1.24–3.41), smoking during pregnancy (1.78; 1.15–2.78), and attending < 6 antenatal care visits (1.61; 1.08–2.40). Factors associated with maternal anemia were (aPR; 95% CI): maternal age < 19 years (1.18; 1.01–1.38), gestational malaria (1.22; 1.01–1.49), not taking micronutrient supplements during pregnancy (1.27; 1.01–1.62), and attending < 6 antenatal care visits (1.40; 1.15–1.70). High prevalence rates of GXN and maternal anemia in these postpartum women may reflect poor assistance during antenatal care, underlying the importance of rethinking current protocols related to nutrition in pregnancy.

## Introduction

Vitamin A deficiency (VAD) and anemia are among the main nutrition-related problems of public health concern worldwide, especially in vulnerable populations in low-resource settings, such as women of childbearing age or preschool children [1–3]. Both VAD and anemia have been considered important determinants of poor maternal and child outcomes, such as impaired growth and development *in utero*, premature delivery, low birth weight, infectious diseases, child development, and maternal and child mortality [2–6].

Depending on its severity, VAD can progress to clinical ocular symptoms, leading to permanent visual impairment if not treated, named xerophthalmia. The first clinical manifestation of xerophthalmia that can be assessed is night blindness, a condition in which one cannot see in low-light conditions [7].

The latest report from the World Health Organization (WHO) estimates that the global prevalence of gestational night blindness (GXN) in populations at risk of VAD at 7.8% (9.75 million pregnant women), and prevalence rates ≥ 5% are considered of public health concern [3]. In Brazil, the public health significance of GXN was considered mild (3.5%); however, the estimates for Brazil were retrieved through regression-based methods, owing to a lack of available data in the country [3]. Also, regional data suggest that prevalence of GXN in the country might be underestimated, as a prevalence of 9.9% was observed in a maternity hospital in Rio de Janeiro city [8].

According to WHO, 41.8% of all pregnancies worldwide are affected by anemia [4], and half of these cases are caused by iron deficiency [2]. The latest official Brazilian data show a prevalence of anemia of 29.4% in women of childbearing age, reinforcing the hypothesis that a considerable number of women will start their pregnancies iron-deficient, increasing the risk of anemia in pregnancy [9].

Among the principal causes of VAD and anemia in pregnancy is the inadequate dietary intake of foods rich in vitamin A (VA) and iron, along with high rates of infection, and aspects related to the physiology of pregnancy, such as the hemodilution in the third trimester [3]. Other underlying causes for VAD and anemia include poor socioeconomic and environmental conditions, maternal illiteracy, history of abortion, and inadequate antenatal care [3,8,10].

Most evidence on GXN and maternal anemia in Brazil does not come from population-based studies, and there is a lack of new studies in less-advantaged areas. Thus, we aimed to describe the prevalence and the factors associated with GXN and maternal anemia in a population-based cross-sectional study in the Brazilian Amazon.

## Methods

### Population and study site

The present cross-sectional analysis refers to the baseline assessment of the MINA-Brazil Study (Maternal and Child Health in Acre, Brazil), which is a large, population-based prospective birth cohort study held in the municipality of Cruzeiro do Sul, Acre State, Western Brazilian Amazon. The municipality of Cruzeiro do Sul has overmore than 80,000 inhabitants, 70% of them living in the urban area. This is the second most populated city in Acre State, situated nearly 640 km west of the state capital, Rio Branco [11]. The 2010 Municipal-level Human Development Index for Cruzeiro do Sul was 0.664 [11]. Only 12.7% of the households in Cruzeiro do Sul had access to proper sanitation in 2010 [11–13]. Furthermore, the municipality accounted for 15.2% of the malaria burden in the country in 2016 (75% due to *Plasmodium* vivax and 25% due to *Plasmodium* falciparum [Ministry of Health of Brazil, unpublished data]), being located in the main malaria endemic-area in Brazil, the Juruá River Valley [14].

Recruitment of participants was conducted at the Women and Children’s Hospital of Juruá Valley, the only maternity ward in the municipality where roughly 96% of all deliveries take place [14,15]. Between July 1^st^, 2015 and June 30^th^, 2016, all delivery-related admissions of women living in Cruzeiro do Sul were identified through daily visits to the maternity ward. The research team visited mothers within the first 12 hours after delivery, before hospital discharge, to explain the study protocol and to invite to participate. Upon acceptance, an interview was held in order to collect data on socioeconomic, environmental, obstetric history, and gestational characteristics. Tablets with CSPro software (https://www.census.gov/programs-surveys/international-programs.html) were used for data-entry. The analysis was restricted to singleton deliveries.

### Outcomes of interest and covariates

The first outcome of interest was GXN, assessed using the standardized interview proposed by WHO and adapted to the Brazilian context [16,17]. This interview is composed of three questions on the subject’s visual capacity in daylight and dim light, taking the visual capacity prior pregnancy as reference, as follows: 1) do you have difficulty seeing during the day? 2) do you have difficulty seeing with decreased light or at night? 3) do you have night blindness? A case of GXN was ascertained when the participant responded NO to the first question and YES to either the second or third question, or both. Women with any visual impairment were asked about their visual ability using glasses or contact lens [17]. To facilitate the comprehension of questions, simple language and examples of places with dim light in the municipality were used [16]. The second outcome assessed was maternal anemia, classified as maternal hemoglobin at delivery < 110.0 g/L at sea level [2]. Venous blood samples were collected and maternal blood hemoglobin was determined by an automated cell counter (Labtest SDH-20, Brazil).

The following covariates were collected and grouped into: socioeconomic—maternal age (< 19 or ≥ 19 years), maternal education (< 9 or ≥ 9 years), maternal skin color (brown, black, white, or indigenous/yellow), living with a partner (no or yes), head of the family (participant or others), maternal occupation (unpaid or paid job), receipt of the *Bolsa Família* conditional cash transfer program (no or yes), ownership of varied assets (no or yes); environmental—water supply (general water supply, water well or river/rain), sanitary facility (septic tank, rudimentary tank or open air/ river), number of residents in the household (≤ 2, 3, 4 or ≥ 5), type of household (masonry or wood/mix [masonry + wood]), area of residence (rural or urban); obstetric history—menarche age (≤ 13 or ≥ 14 years), history of fetal loss (no or yes), primigravidae (no or yes); and gestational characteristics—smoking during pregnancy (no or yes), gestational urinary tract infection (no or yes), nutritional supplement use (none, iron + folic acid, or multiple micronutrients with VA), number of antenatal care visits (< 6, 6–8, or ≥ 9), gestational malaria (no or yes), and gestational age at delivery (weeks).

In order to assess the socioeconomic status of the participant’s household, a wealth index was created using principal component analysis, according to the presence of household assets [18]. The first principal component explained 22.8% of the variability between the households. Scores for each asset were summed up, giving an index of household wealth, consequently split into quintiles (the 1^st^ quintile corresponds to the poorest households and the 5^th^ to the wealthiest households).

Data on gestational urinary tract infection, nutritional supplement use, and the number of antenatal care appointments were checked in each participant’s antenatal care registries. Gestational malaria episodes were retrospectively obtained from the Malaria Epidemiological Surveillance and Information System (SIVEP) database from the Ministry of Health of Brazil (http://200.214.130.44/sivep_malaria/). The final gestational age was consulted from the hospital records.

### Sub-sample analysis

Among participants recruited at the maternity ward, a sub-sample of pregnant women (n = 528) who were attending public antenatal care clinics in the urban area of the municipality were followed-up since the antenatal period. They were recruited for the study with 20 weeks of pregnancy or less, based on their last menstrual period, and invited for two clinical assessments at 16–20 weeks and at about 28 weeks of pregnancy.

Blood samples were collected in each clinical assessment and shipped to analysis in São Paulo, according to previous protocols used in similar field conditions [19]. Serum concentrations of retinol were measured using high performance liquid chromatography (HP-1100 HPLC system, Hewlett Packard, Palo Alto, California, USA) [20], and the values categorized followed WHO recommendations (< 0.7 μmol/L–deficiency; 0.7–1.05 μmol/L–insufficiency; ≥ 1.05 μmol/L–sufficiency) [3]. After this, we further explored the VA status by creating two variables deeming the occurrence of VAD and VA insufficiency combining results for both antenatal assessments, independently of their occurrence: combined VA status–deficiency (not deficient during pregnancy or deficient during pregnancy) and combined VA status–insufficiency (sufficient during pregnancy or insufficient during pregnancy). We also explored the variation of serum retinol concentrations between assessments (serum retinol in the 2^nd^ assessment minus serum retinol in the 1^st^ assessment) as ‘tertiles of the difference of serum retinol’. Plasma ferritin concentrations were measured by enzyme immunoassays (Ramco, Houston, TX) and the cut off adopted for iron deficiency was < 15 μg/L [2]. Plasma C-reactive protein (mg/L) was measured by an IMMAGE Immunochemistry System (Beckman Coulter, Brea, CA, USA), adopting concentrations ≥ 5 mg/L as acute inflammation [21]. Plasma ferritin and C-reactive protein were only available in the 2^nd^ assessment (n = 464). Intra- and inter-assay coefficient of variation were < 2.5% and 7%, respectively.

### Statistical analysis

Frequencies were used to characterize the sample, and the Pearson’s and Wald’s chi-square tests were used to compare the differences between proportions. The same approach was used for the sub-sample analysis. Multiple Poisson regression models with robust variance through a hierarchical selection of covariates were used [22]. A hierarchical conceptual model considered four levels of determination, as follows: 1—socioeconomic; 2—environmental; 3—obstetric history; 4—gestational characteristics. Covariates associated with each outcome at p ≤ 0.20 in crude analysis, or in view of their significance in the literature, were selected to multiple models. At each level of determination, covariates associated with outcome at p < 0.10 or if conceptually relevant were retained for the final multiple model. Missing observation (<5%) were included in the models by creating missing-value categories. Associations with the outcomes are presented with unadjusted and adjusted prevalence ratios (aPR) and respective 95% confidence intervals (95% CI). All p-values were derived from two-tailed tests. Stata 15 (StataCorp, College Station, Texas, USA) was used to perform the analysis.

### Details of ethical approval

The MINA-Brazil Study was approved by the Ethics Committee of School of Public Health, University of São Paulo (Protocol number 872.613, Nov 13^th^, 2014), and by Acre State Health Secretary. The consent to participate was given through signature of the written informed consent. In case of teenage parturients consent was obtained from the caregiver. For cases of illiteracy, a thumb finger print was obtained.

## Results

### Characteristics of the study population

**Fig 1:**
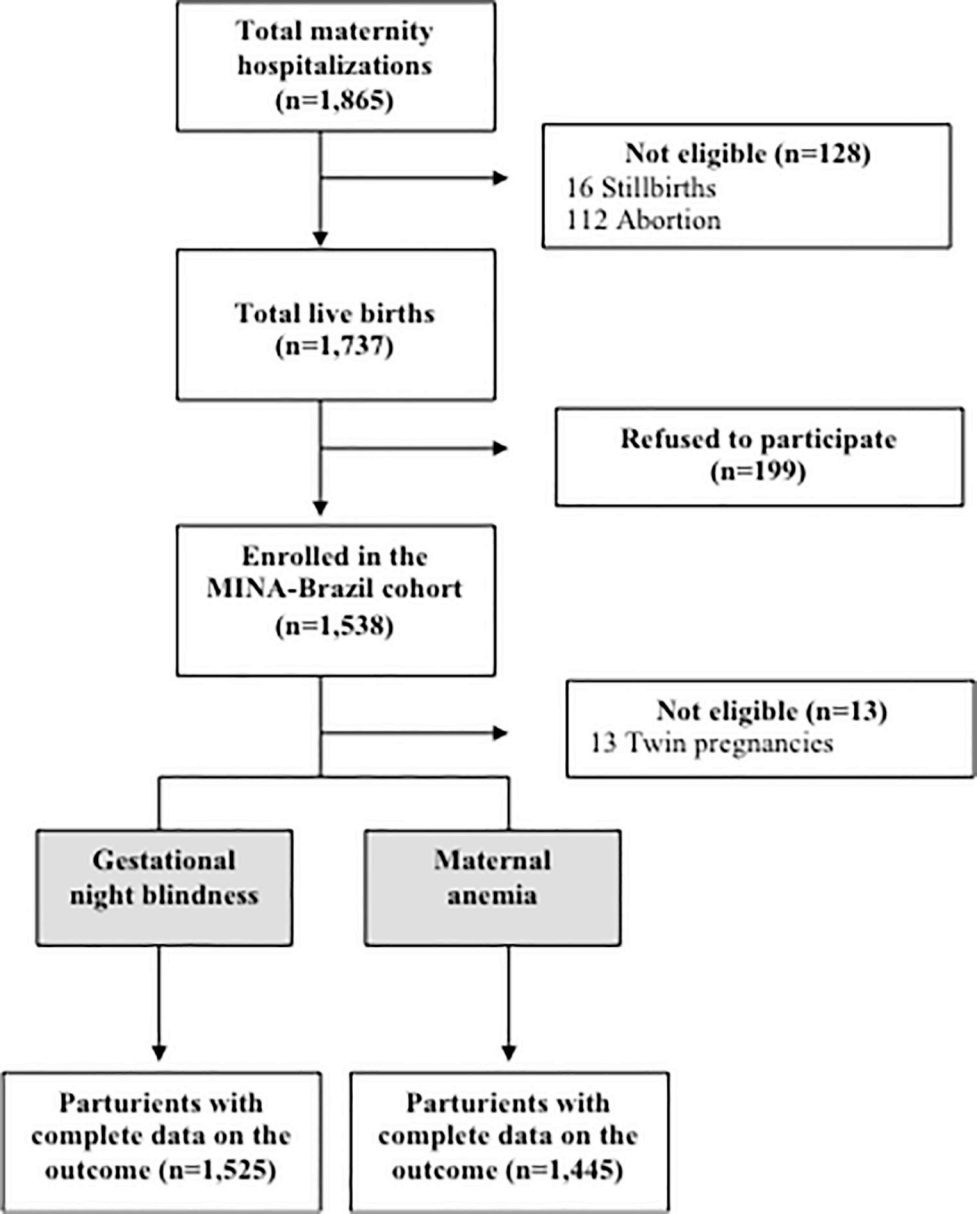
Flow-diagram of screening of participants and outcome assessment.

During the recruitment period, 1,865 hospitalizations of women living in Cruzeiro do Sul were registered, of which 128 were not eligible (16 stillbirths and 112 abortions). The remaining 1,737 women were admitted for delivery; of these 1,538 gave consent and were enrolled in the study. Out of 1,538 postpartum women who agreed to participate in the study, 1,525 (99.2%) had data on GXN, and 1,445 (94.0%) had data on maternal anemia. Additional exclusions (n = 13) occurred owing to twin pregnancies (**Fig 1**).

In **Table 1**, socio-demographic and environmental characteristics of participants are shown. About 20% of parturients were adolescents (< 19 years) and more than 40% of them had less than 9 years of formal schooling. More than three quarters of postpartum women reported ‘brown’ as their skin color, with only 11.7% reporting ‘white’ (data not shown in Tables). The obstetrics and gestational characteristics of participants are given in **Table 2**. The majority of participants took supplements during pregnancy (90.5%) and attended more than six antenatal care visits (72.4%).

**Table 1:** Socio-demographic and environmental characteristics of parturients from the MINA-Brazil study by outcome.

Variables	Total^c^	n (%)	GXN^d^ (n = 176)		Anemia (n = 569)	
			n (%)	P	n (%)	P
Maternal age^a^	1,525			0.070		<0.001
< 19 years		303 (19.9)	44 (14.5)		145 (49.8)	
≥ 19 years		1,222 (80.1)	132 (10.8)		424 (36.7)	
Maternal schooling^a^	1,477			0.001		0.005
< 9 years		626 (42.4)	91 (14.8)		254 (43.4)	
≥ 9 years		851 (57.6)	76 (9.0)		294 (36.0)	
Living with a partner^a^	1,478			0.714		0.035
No		320 (21.6)	34 (10.8)		135 (44.2)	
Yes		1,158 (78.4)	133 (11.6)		413 (37.1)	
Head of the family^a^	1,478			0.133		0.094
Woman		195 (13.2)	16 (8.2)		63 (33.5)	
Others		1,283 (86.8)	151 (11.9)		485 (39.9)	
Beneficiary of conditional cash transfer program^a^	1,478			0.004		0.035
No		841 (56.9)	78 (9.4)		295 (36.7)	
Yes		637 (43.1)	89 (14.2)		253 (42.2)	
Wealth Index (quintiles)^b^	1,478			0.076		0.011
1		294 (19.9)	40 (13.7)		116 (42.3)	
2		294 (19.9)	37 (12.8)		121 (43.2)	
3		297 (20.1)	29 (9.9)		109 (39.1)	
4		295 (20.0)	34 (11.6)		105 (37.1)	
5		298 (20.1)	27 (9.1)		97 (33.8)	
Sanitary facility^b^	1,478			0.030		0.022
Septic tank		732 (49.5)	69 (9.5)		248 (35.6)	
Rudimentary tank		320 (21.7)	42 (13.2)		130 (43.0)	
Open air/ river		426 (28.8)	56 (13.4)		170 (42.0)	
Number of people in the household^b^	1,478			<0.001		0.003
≤ 2		296 (20.0)	19 (6.5)		99 (35.2)	
3		348 (23.5)	29 (8.4)		114 (34.2)	
4		266 (18.0)	32 (12.1)		101 (39.0)	
≥ 5		568 (38.5)	87 (15.5)		234 (44.0)	
Type of household^a^	1,478			0.086		0.264
Masonry		381 (25.8)	34 (9.0)		134 (36.6)	
Wood/ mix (masonry + wood)		1,097 (74.2)	133 (12.3)		414 (39.9)	

^a^Chi-squared test p-values

^b^Wald test p-values for trend

^c^Totals differ from the total number of participants due to missing values

^d^Gestational night blindness.

**Table 2:** Obstetric and gestational characteristics of parturients from the MINA-Brazil study by outcome.

Variables	Total^d^	n (%)	GXN^e^ (n = 176)		Anemia (n = 569)	
			n (%)	P	n (%)	P
Primigravidae^a^	1,478			0.917		0.025
No		887 (60.0)	101 (11.5)		308 (36.6)	
Yes		591 (40.0)	66 (11.3)		240 (42.6)	
History of fetal losses^a^	877			0.185		0.622
No		612 (69.0)	64 (10.5)		208 (36.1)	
Yes		275 (31.0)	37 (13.6)		100 (37.8)	
Smoking during pregnancy^a^	1,478			<0.001		0.838
No		1,415 (95.7)	151 (10.8)		525 (39.0)	
Yes		63 (4.3)	16 (25.8)		23 (40.3)	
Gestational urinary tract infection^a^	1,525			0.333		0.121
No		544 (35.7)	57 (10.5)		189 (36.7)	
Yes		981 (64.3)	119 (12.1)		380 (40.8)	
Gestational supplementation^b^	1,524			0.125		<0.001
None		145 (9.5)	22 (15.1)		64 (47.4)	
Iron + folic acid		890 (58.4)	104 (11.7)		351 (41.6)	
Multiple micronutrients with vitamin A		489 (32.1)	50 (10.2)		154 (33.0)	
Maternal anemia at delivery^a^^,^^c^	1,445			0.024		
No		876 (60.6)	85 (9.7)		-	-
Yes		569 (39.4)	77 (13.5)		-	-
Gestational night blindness^a^	1,525					0.024
No		1,349 (88.5)	-	-	492 (38.3)	
Yes		176 (11.5)	-	-	77 (47.5)	
Number of antenatal care appointments^b^	1,503			<0.001		<0.001
< 6		415 (27.6)	69 (16.6)		177 (45.6)	
6–8		655 (43.6)	67 (10.2)		260 (42.0)	
≥ 9		433 (28.8)	37 (8.5)		123 (29.5)	
Gestational malaria^a^	1,525			0.759		0.053
No		1,404 (92.1)	161 (11.4)		514 (38.6)	
Yes		121 (7.9)	15 (12.4)		55 (47.8)	

^a^Chi-squared test p-values

^b^Wald test p-values for trend

^c^ Hemoglobin at delivery < 110 g/L

^d^Totals differ from the total number of participants due to missing values

^e^Gestational night blindness.

### Prevalence of the outcomes and association with nutrient biomarkers in pregnancy

The prevalence of GXN was 11.5% (95% CI 9.97–13.25, n = 176) and maternal anemia was detected in 39.4% (95% CI 36.84–41.95, n = 569) of participants at delivery (**Table 2**). Mean hemoglobin concentration at delivery was 111.67 g/L (95% CI 110.96–112.38). Almost half of the women with GXN concomitantly presented maternal anemia (47.5%, n = 77, data not shown in Tables).

In the sub-sample analysis, VA deficiency in the first and second assessments affected 10.6% and 6.4% of participants, respectively (**S1 Table**). Median values for serum retinol were of 1.77 μmol/L (1.00–2.60) in the first assessment and 1.9 μmol/L (1.2–2.7) in the second one. The VA status in the 1^st^ assessment and the combined VAD in the antenatal period were associated significantly with GXN. In addition, VA status in the 1^st^ assessment, as well as combined VAD, combined VA insufficiency, and iron deficiency were associated with maternal anemia (**S1 Table**). The occurrence of acute inflammation was only 0.2% (data not shown in Tables).

### Factors associated with GXN and maternal anemia

**Table 3** presents the crude and adjusted analysis of factors associated with GXN. In crude analysis, socioeconomic variables (wealth index, beneficiary of cash transfer program, maternal schooling and number of people in the house) were associated to higher risk for gestational night blindness when compared to reference strata for better life conditions. These associations were attenuated after multiple adjustment in the same distal determination level, and they were kept in the final fully adjusted model following hierarchical selection of independent variables. After controlling for potential confounders, parturients who were living in a household with four or more residents presented a higher prevalence rate of GXN when compared with those sharing the household with fewer members (five or more—aPR 2.06, 95% CI 1.24–3.41). Smoking during pregnancy was associated with an increased prevalence rate of GXN (aPR 1.78, 95% CI 1.15–2.78) in comparison with non-smokers. Participants who completed insufficient antenatal care visits (<6) had higher prevalence rate of GXN when compared with those who had more than 9 appointments (aPR 1.61, 95% CI 1.08–2.40) (**Table 3**).

**Table 3:** Unadjusted and adjusted analysis of associated factors with gestational night blindness.

Variables	n	GXN^b^	
	1,525	PR (95% CI)^c^	aPR (95% CI)^d^
Wealth Index (quintiles)			
5		Reference	Reference
4		1.33 (0.82; 2.16)	1.08 (0.66; 1.75)
3		1.23 (0.75; 2.02)	0.82 (0.49; 1.38)
2		1.43 (0.88; 2.31)	0.97 (0.59; 1.60)
1		1.56 (0.98; 2.49)	0.88 (0.51; 1.49)
Beneficiary of conditional cash transfer program			
No		Reference	Reference
Yes		1.50 (1.13; 2.00)	1.08 (0.79; 1.48)
Maternal schooling			
≥ 9 years		Reference	Reference
< 9 years		1.63 (1.22; 2.17)	1.25 (0.90; 1.74)
Number of people in the household			
≤ 2		Reference	Reference
3		1.29 (0.74; 2.26)	1.36 (0.76; 2.44)
4		1.86 (1.08; 3.20)	1.82 (1.02; 3.22)
≥ 5		2.38 (1.48; 3.84)	2.06 (1.24; 3.41)
History of fetal losses			
No		Reference	Reference
Yes		1.29 (0.88; 1.88)	1.36 (0.94; 1.96)
Gestational supplementation			
Multiple micronutrients with vitamin A		Reference	Reference
Iron + folic acid		1.14 (0.83; 1.57)	0.92 (0.67; 1.28)
None		1.48 (0.93; 2.36)	1.01 (0.61; 1.68)
Postpartum maternal anemia^a^			
No		Reference	Reference
Yes		1.39 (1.04; 1.86)	1.26 (0.94; 1.68)
Smoking during pregnancy			
No		Reference	Reference
Yes		2.38 (1.52; 3.73)	1.78 (1.15; 2.78)
Number of antenatal care appointments			
≥ 9		Reference	Reference
6–8		1.19 (0.81; 1.75)	1.11 (0.75; 1.63)
< 6		1.94 (1.33; 2.83)	1.61 (1.08; 2.40)

^a^Hemoglobin at delivery < 110 g/L

^b^Gestational night blindness

^c^Unadjusted prevalence ratio with respective 95% confidence interval

^d^Adjusted prevalence ratio with respective 95% confidence interval.

Crude and adjusted analysis of factors associated with maternal anemia are presented in **Table 4**. Likewise the associated factors for gestational night blindness, the lower quintiles of wealth index were associated to higher risk for maternal anemia in crude analysis. These associations were attenuated after multiple adjustment following hierarchical selection of independent variables. After multiple adjustment, teenager postpartum women were more likely to be anemic at delivery than adult parturients (aPR 1.18, 95% CI 1.01–1.38). An inverse gradient was seen for the number of antenatal care appointments, as a higher prevalence rate of anemia was observed among women who completed insufficient visits (<6: aPR 1.40, 95% CI 1.15–1.70). Malaria occurrence during pregnancy was associated with maternal anemia in this population (aPR 1.22, 95% CI 1.01–1.49). Compared with multiple micronutrients use, not taking any nutritional supplement during pregnancy was associated with higher prevalence of maternal anemia (aPR 1.27, 95% CI 1.01–1.62), whereas the use of iron-folic acid supplement was not significantly associated (**Table 4**). Further adjustment for gestational age at delivery did not change the associations observed for both outcomes.

**Table 4:** Unadjusted and adjusted analysis of associated factors with postpartum maternal anemia.

Variables	n	Anemia
	1,445	PR (95% CI)^a^	aPR (95% CI)^b^
Wealth Index (quintiles)			
5		Reference	Reference
4		1.02 (0.81; 1.28)	0.99 (0.79; 1.24)
3		1.19 (0.96; 1.47)	0.99 (0.79; 1.24)
2		1.23 (0.99; 1.52)	1.09 (0.87; 1.36)
1		1.24 (1.00; 1.53)	1.02 (0.81; 1.29)
Maternal age			
≥ 19 years		Reference	Reference
< 19 years		1.35 (1.18; 1.55)	1.18 (1.01; 1.38)
Number of people in the household			
≤ 2		Reference	Reference
3		0.96 (0.77; 1.19)	1.00 (0.80; 1.26)
4		1.10 (0.88; 1.38)	1.09 (0.87; 1.36)
≥ 5		1.23 (1.02; 1.48)	1.16 (0.96; 1.41)
Primigravidae			
No		Reference	Reference
Yes		1.15 (1.00; 1.31)	1.10 (0.94; 1.29)
Gestational urinary tract infection			
No		Reference	Reference
Yes		1.11 (0.97; 1.27)	1.14 (0.99; 1.30)
Gestational night blindness			
No		Reference	Reference
Yes		1.23 (1.03; 1.47)	1.14 (0.95; 1.35)
Gestational malaria			
No		Reference	Reference
Yes		1.27 (1.02; 1.52)	1.22 (1.01; 1.49)
Gestational supplementation			
Multiple micronutrients with vitamin A		Reference	Reference
Iron + folic acid		1.26 (1.08; 1.47)	1.15 (0.98; 1.35)
None		1.43 (1.15; 1.79)	1.27 (1.01; 1.62)
Number of antenatal care appointments			
≥ 9		Reference	Reference
6–8		1.42 (1.19; 1.69)	1.37 (1.15; 1.64)
< 6		1.54 (1.28; 1.85)	1.40 (1.15; 1.70)

^a^Unadjusted prevalence ratio with respective 95% confidence interval

^b^Adjusted prevalence ratio with respective 95% confidence interval.

## Discussion

High prevalence rates of GXN and maternal anemia were found in this population-based study in Western Brazilian Amazon. The prevalence of GXN (11.54%) was more than twice the WHO cut-off for public health significance in pregnancy [3]. The prevalence of maternal anemia affected almost 40% of the participants. GXN was associated with VAD during pregnancy, regardless of the time of assessment, as well as with the number of residents living in the same household, smoking during pregnancy, and the insufficient number of antenatal care appointments. Maternal anemia was associated with almost all biomarkers of VA and iron status measured in the sub-sample analysis, and with maternal age, the insufficient number of antenatal care visits, malaria infection and nutritional supplement use in pregnancy.

The population basis of our study allowed us to obtain estimates of prevalence rates that are representative of this low-resource study area. However, even though the interview used for assessment of GXN is a standardized instrument proposed by WHO, and adequate for epidemiological purposes, the method used relies on the respondents’ memory recall and ability to understand the questions. Although associations of GXN with VAD in pregnancy were confirmed regardless of the time of assessment in our sub-sample analysis, a recall bias cannot be discharged. The lack of a local term to describe the symptom may also have hindered comprehension [7].

Caution is needed in the interpretation of our results considering the limitations of the tool used to assess GXN. Historically, the assessment of night blindness by the WHO questionnaire has been one of the most commonly used and recommended methods for assessing VAD in vulnerable populations, even though the reported tool does not correlate strongly with serum retinol [7,23], likely owing to the hepatic regulation of this biomarker when retinol stores are replete in the liver [24]. Nevertheless, this is a low-cost, less-invasive method to monitor VA status in populations. In this study, the gestational VAD assessed by low serum retinol concentrations were associated significantly with GXN, supporting the use of WHO questionnaire in this population.

Global estimates of night blindness in women of childbearing age, retrieved from the WHO Global Database on Vitamin A Deficiency [25], reveal important prevalence among some selected countries, especially in Asia, although the last update occurred more than 10 years ago. Prevalence rates less than 10% were registered in Indonesia (1.7%), Madagascar (7.5%), and Nigeria (7.7%), whereas high prevalence rates were observed in Bolivia (14.0%), Nepal (19.6%), and in the district of Medak, India (35.0%) [25]. There are no countrywide data for GXN in Brazil; most investigations have been concentrated in Rio de Janeiro, where a prevalence of GXN of 9.9% was found in a maternity hospital [8]. This result aligns with our findings in Brazilian Amazon. Moreover, the last Demographic Health Survey conducted in Brazil showed that 12.3% of women of childbearing age were VA deficient [9].

Poor social, environmental, and pregnancy-related factors conditions have been associated with GXN [3,26]. In rural Nepal, a population-based case-control study found that patients with GXN were more likely to be illiterate, to be from lower castes, and to live in households of poor quality [26]. Similarly, in a population-based trial of newborn VA supplementation in rural India, having a concrete roof, literacy, and being pregnant during adulthood lowered the odds of GXN, whereas higher parity increased the odds [10]. A cross-sectional study in a maternity ward in Rio de Janeiro city found that living in wealthier areas of the city and receiving antenatal nutritional assistance reduced the odds for GXN, whereas having a history of abortion and having anemia during pregnancy increased the odds [8]. Counselling during antenatal care may act on the improvement of lifestyle behaviors, like quitting smoking and increasing the intake of nutrient-rich foods, especially in VA. Pregnant women who live in a household with a high number of residents are likely to share their home resources, lowering food availability to supply their own physiological needs during pregnancy.

The WHO global estimates indicate that anemia in pregnancy is a worldwide issue, and almost all countries had prevalence rates higher than 20% in 2011 [27]. In this report, estimates for Brazil set anemia in pregnancy as a moderate public health problem, with a prevalence rate of 32% (95% CI 11–62) and mean hemoglobin of 116.0 g/L (95% CI 106–128). In our study, prevalence of anemia in parturients was even higher than those estimated by the WHO for Brazil, comparable with rates for Asia [27], where gestational malaria can be also one of the associated factors with maternal anemia.

Classical factors associated with anemia include iron deficiency, due to low consumption, higher needs or inadequate absorption, other micronutrient deficiencies (e.g. folate and VA), infections (e.g. malaria and HIV), and hemoglobinopathies [19,27]. The iron-folic acid supplementation is a recognized intervention to reduce the anemia burden in pregnancy [4,28]. In our study, anemia was associated with being a teenage parturient, presenting malaria episodes, attending insufficient antenatal care visits, and not using nutritional supplements in pregnancy. Nutritional demands are higher for teenagers than for adult women as they are still undergoing physiological development, and pregnancy in adolescence exacerbates such demands by concomitantly needs for the fetus growth [10]. Malaria is a proven risk factor for anemia in pregnancy, because the rupture of red blood cells during *Plasmodium* parasite’s metabolism lowers the red blood cell count [29]. During antenatal care, interventions aimed to prevent anemia are advised, such as encouraging on intake of iron-rich foods and iron-folic acid supplementation [4].

Noteworthy, such high prevalence of GXN and maternal anemia in this study ascertain the importance of achieving a good nutritional status and health assistance during pregnancy in a context of social and environmental vulnerability [12,29]. The current nutrition strategies recommended by the WHO for targeting micronutrient deficiencies in pregnancy include supplementation with only iron and folic acid [4]. Recently, multiple micronutrients supplementation has been proven effective to prevent of harmful effects of micronutrient deficiencies in pregnancy [30]. Taking into account the positive results for multiple micronutrients and the synergy among micronutrients [31], the WHO nutrition-related protocols for antenatal care should be reviewed.

## Conclusion

GXN and maternal anemia were identified as significant public health problems in the Brazilian Amazon municipality studied. Such high prevalence of GXN and maternal anemia may reflect the poor nutritional status experienced by these women prior and during pregnancy, which can have deleterious lifetime effects for the dyad mother-infant. Sociodemographic, environmental and gestational characteristics were associated with the occurrence of GXN and maternal anemia. Most of these factors can be addressed during antenatal care, underlying the importance of rethinking current protocols related to nutrition in pregnancy.

## Supporting information

S1 Table Vitamin A and iron status during pregnancy according to gestational night blindness (GXN) and anemia among participants of the MINA-Brazil study. (DOCX)

## Abbreviations

aPR: adjusted prevalence ratio
GXN: gestational night blindness
VA: vitamin A
VAD: vitamin A deficiency
WHO: World Health Organization
