# Supplementary material for: High prevalence of gestational night blindness and maternal anemia in a population-based survey of Brazilian Amazonian postpartum women
Source: PLoS One. 2019 Jul 3;14(7):e0219203. doi: 10.1371/journal.pone.0219203 (PMC6608963; doi:10.1371/journal.pone.0219203)
Supplement: S1 Table — (DOCX) [file pone.0219203.s001.docx]

S1 Table. Vitamin A and iron status during pregnancy according to gestational night blindness (GXN) and anemia among participants of the MINA-Brazil study.

| Variables | Total^f^ | n (%) | GXN | |  | Anemia | |
| --- | --- | --- | --- | --- | --- | --- | --- |
|  |  |  | n (%) | P |  | n (%) | P |
| Vitamin A status in the 1^st^ assessment^a,b^ | 518 |  |  | 0.006 |  |  | 0.009 |
| ≥ 0.7 µmol/L |  | 463 (89.4) | 26 (6.21) |  |  | 146 (35.2) |  |
| < 0.7 µmol/L |  | 55 (10.6) | 9 (16.7) |  |  | 28 (53.8) |  |
| Vitamin A status in the 2^nd^ assessment^a,c,d^ | 467 |  |  | 0.461 |  |  | 0.120 |
| ≥ 0.7 µmol/L |  | 437 (93.6) | 30 (7.4) |  |  | 147 (36.8) |  |
| < 0.7 µmol/L |  | 30 (6.4) | 3 (10.7) |  |  | 14 (51.8) |  |
| Combined vitamin A status – insufficiency^a,e^ | 451 |  |  | 0.711 |  |  | 0.025 |
| No insufficiency during pregnancy |  | 260 (57.6) | 14 (7.7) |  |  | 76 (32.5) |  |
| Insufficient during pregnancy |  | 191 (42.4) | 16 (6.7) |  |  | 77 (43.2) |  |
| Combined vitamin A status – deficiency^a,e^ | 451 |  |  | 0.017 |  |  | 0.007 |
| No deficiency during pregnancy |  | 375 (83.2) | 20 (5.8) |  |  | 117 (34.2) |  |
| Deficient during pregnancy |  | 76 (16.8) | 10 (13.7) |  |  | 36 (51.4) |  |
| Iron status (serum ferritin)^a,c^ | 464 |  |  | 0.794 |  |  | 0.002 |
| < 15 µg/L |  | 197 (42.5) | 14 (7.6) |  |  | 76 (31.5) |  |
| ≥ 15 µg/L |  | 267 (57.5) | 17 (6.9) |  |  | 85 (46.4) |  |

^a^Chi-squared test p-values;

^b^Between 16 to 20 weeks of pregnancy;

^c^Fisher’s exact test;

^d^About 28 weeks of pregnancy;

^e^For ‘*insufficient during pregnancy*’ was considered insufficiency (serum retinol < 1.05 µmol/L) in at least one assessment or both; for ‘*deficient during pregnancy*’ was considered deficiency (serum retinol < 0.7 µmol/L) in at least one assessment or both;

^f^Totals differ from the total number of participants studied because of missing values.
